# Histomorphometry Changes and Decreased Reactivity to Angiotensin II in the Ileum and Colon of Streptozotocin-Induced Diabetic Rats

**DOI:** 10.3390/ijms232113233

**Published:** 2022-10-31

**Authors:** Marisa Esteves-Monteiro, Daniela Menezes-Pinto, Mariana Ferreira-Duarte, Patrícia Dias-Pereira, Manuela Morato, Margarida Duarte-Araújo

**Affiliations:** 1 LAQV-REQUIMTE, Faculty of Pharmacy, University of Porto, 4050-313 Porto, Portugal; 2 Department of Immuno-Physiology and Pharmacology, Institute of Biomedical Sciences Abel Salazar, University of Porto (ICBAS-UP), 4050-313 Porto, Portugal; 3 Laboratory of Pharmacology, Department of Drug Sciences, Faculty of Pharmacy, University of Porto (FFUP), 4050-313 Porto, Portugal; 4 Department of Pathology and Molecular Immunology, Institute of Biomedical Sciences Abel Salazar, University of Porto (ICBAS-UP), 4050-313 Porto, Portugal

**Keywords:** diabetes mellitus, STZ, ileum histomorphometry, colon histomorphometry, smooth muscle contraction, Angiotensin II receptors

## Abstract

Diabetes mellitus (DM) is a chronic progressive metabolic disorder associated with several gastrointestinal complications, affecting up to 75% of patients. Knowing that Angiotensin II (AngII) also regulates intestinal contraction, we decided to evaluate changes in ileum and colon histomorphometry and AngII reactivity in a rat model of DM. Streptozotocin (STZ, 55 mg/kg) was administered to induce DM to 24 adult male Wistar rats. Diabetic rats displayed all the characteristic signs of type 1 DM (T1DM) and fecal excretion increased about 4-fold over 14 days, while the excretion of controls remained unaltered. Compared to controls, diabetic ileum and colon presented an increase in both macroscopic (length, perimeter and weight) and microscopic (muscular wall thickness) parameters. Functionally, AngII-induced smooth muscle contraction was lower in diabetic rats, except in the distal colon. These differences in the contractile response to AngII may result from an imbalance between AngII type 1 (antagonized by candesartan, 10 nM) and type 2 receptors activation (antagonized by PD123319, 100 nM). Taken together, these results indicate that an early and refined STZ-induced T1DM rat model already shows structural remodelling of the gut wall and decreased contractile response to AngII, findings that may help to explain diabetic dysmotility.

## 1. Introduction

Diabetes mellitus (DM) is a complex chronic progressive metabolic disorder, medically incurable, that can affect almost every organ system [1]. There are different animal models of DM, but streptozotocin (STZ) has been the agent of choice to chemically induce diabetes in rats and mice, causing the selective destruction of pancreatic β-cells. High doses of STZ are associated with type 1 DM (T1DM) induction, while multiple low doses are usually associated with a high fat diet to cause insulin resistance, characteristic of type 2 DM (T2DM) [2,3,4]. In this animal model of T1DM structural, functional and biochemical alterations resemble those observed in human diabetic patients [5]. Over time, several investigators have used this model with different induction times (raising questions about animal welfare for longer protocols) in different portions of the intestine, making it harder to compare results [6,7,8]. For that reason, we decided to assess whether two weeks is sufficient to induce ileum and colon alterations that resemble those observed in long-lasting STZ models [9,10].

Gastrointestinal (GI) complications of DM are very important as they can be associated with significant morbidity, affecting up to 75% of patients [11]. The most common GI complications include esophageal dysmotility, gastroparesis, enteropathy and colonic disorders, such as chronic constipation and diarrhea [7,12]. Since these symptoms are not considered important causes of mortality in patients with DM they are often neglected [13]. However, it is important to recognize that they negatively influence health status and quality of life [13,14].

The pathogenesis of diabetic intestinal dysfunction seems to be multifactorial, related to the accumulation of advanced glycation end-products (AGE), injury of the enteric nervous system (ENS) or interstitial cells of Cajal, and muscular layers fibrosis [8]. Several studies also indicate that diabetic autonomic neuropathy causes damage to the ENS and changes the number and size of myenteric neurons throughout the entire GI tract in rats [15,16,17,18,19,20]. It has also been described as a deficit in the intestine’s cholinergic neurotransmission, since the response to exogenous acetylcholine (ACh) seems to be impaired in the ileum (30 days after STZ-induction) and colon of long-term diabetic rats (60 weeks) [21,22]. Mechanical factors can also contribute to intestinal disorders, since DM seems to cause structural remodeling that can affect histomorphometry and biomechanical properties, increasing stiffness, and decreasing the resting compliance and relaxation capacity of the intestinal wall [9,10,23].

The renin–angiotensin system (RAS) is mostly known for its effects in the cardiovascular and renal systems but it also has an influence in other systems, such as the GI tract, which expresses all of the RAS components [24,25]. Angiotensin II (Ang II) is the major effector peptide of this system, and most of its functions are mediated by the Ang II type 1 receptor (AT_1_R), while activation of the Ang II type 2 receptor (AT_2_R) usually counteracts them [26,27]. In the colon, Ang II contracts circular and longitudinal smooth muscle in response to direct activation of post-junctional AT_1_R and indirect activation of pre-junctional AT_1_R in myenteric and submucosal neurons [26,27,28,29]. Curiously, the human colonic smooth muscle is more sensitive to Ang II than to acetylcholine (ACh), but the physiological importance of Ang II in the GI tract is still not completely understood [25,30,31]. Interestingly, there is little information on RAS alterations in the intestine of diabetic individuals, but recently one study concluded that ACE gene polymorphism in patients with T2DM influences intestinal motility, since those patients presented a prevalent genotype that was associated to constipation [32].

Considering the above, the aim of this study was to evaluate the structural (macro and microscopic histomorphometry) and functional (smooth muscle reactivity to Ang II) impact of T1DM in the ileum and colon of a refined rat model, just two weeks after induction. 

## 2. Results

### 2.1. Animal Welfare and Monitorization

STZ-induced rats had an initial glycemia of 99.30 ± 3.29 mg/dL that increased to 395.09 ± 13.80 mg/dL within 48 h (*p* < 0.0001, *n* = 23), while control rats had an initial glycaemia of 105.63 ± 6.31 mg/dL that was roughly the same within 48 h (111.14 ± 5.41 mg/dL; *p* > 0.05, *n* = 8). On d7 and d14, almost all STZ rats had glycemia above 500 mg/dL, while control animals presented glycemic values of 105.57 ± 4.76 mg/dL (*n* = 8) on the 14th day. 

**Figure 1:**
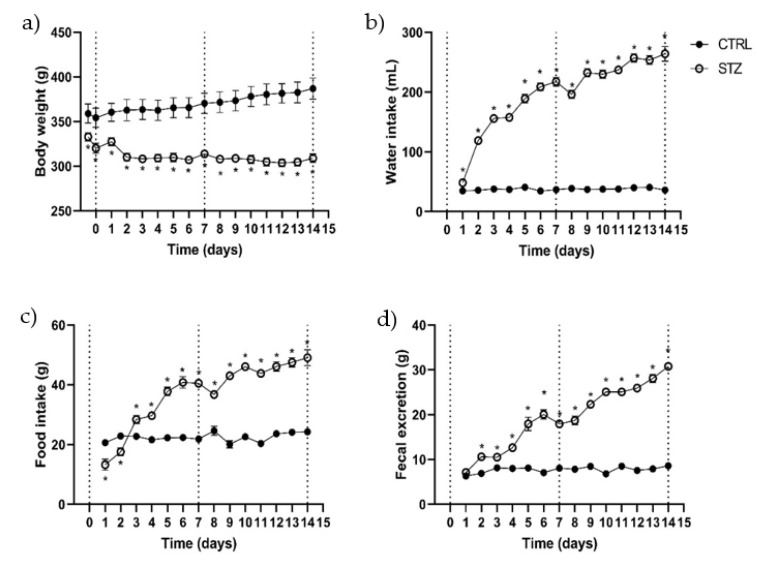
Evaluation during the experimental protocol (14 days) in control (CTRL, *n* = 8) and streptozotocin-induced diabetic rats (STZ, *n* = 16–21) of: (**a**) body weight; (**b**) water intake; (**c**), food intake and (**d**) fecal excretion. Values are mean ± SEM and unpaired student’s *t* test was used to compare the two experimental groups (CTRL and STZ). * Statistical difference, *p* < 0.05.

The parameters documented during the daily monitorization (body weight, water/food intake and fecal excretion) are shown in Figure 1. In the control group (*n* = 8), rats progressively gained weight, their weight being 7.8% ± 0.73% higher by d14 than on d0 (before fasting). Diabetic rats (*n* = 21) had a consistent weight loss that was more pronounced on d2 (5% less compared to the previous day) and then maintained that weight for the remainder of the protocol (7.66 ± 1.04% lower at d14 when compared to the initial weight before fasting) (Figure 1a). Water intake was significantly higher in diabetic rats comparing to controls that maintained a constant water intake through all the experimental protocol: 37.54 ± 0.53 mL/day (*n* = 8). The STZ group drank more water since d1 (48.38 ± 1.16 mL), but their water intake increased progressively throughout the protocol, reaching values 7 times higher than those of control animals at d14: 264.08 ± 12.18 mL (*n* = 16) (Figure 1b). Despite the weight loss, STZ rats’ food intake was significantly higher than controls after the 3rd day. Diabetic rats started the experimental protocol eating 13.25 ± 1.86 g in the first day, and progressively increased food consumption until the last day, when the intake was 49.08 ± 2.64 g/rat (*n* = 16). The control group maintained a constant food intake during the experimental time, with a mean consumption of 22.44 ± 0.38 g/day (*n* = 8) (Figure 1c). 

To our knowledge, this is the first study to quantify fecal excretion in STZ-induced diabetic animals. Non-diabetic animals maintained a relatively stable fecal excretion during the entire experimental period (7.75 ± 0.18 g/day/rat, *n* = 8), whereas diabetic rats gradually increased their fecal excretion, reaching values 4 times higher than those obtained in the first day (d1: 7.11 ± 0.34 g/rat; d14: 30.79 ± 0.73 g/rat; *p* < 0.0001, *n* = 16) (Figure 1d).

### 2.2. Ileum and Colon Macroscopic Evaluation

**Figure 2:**
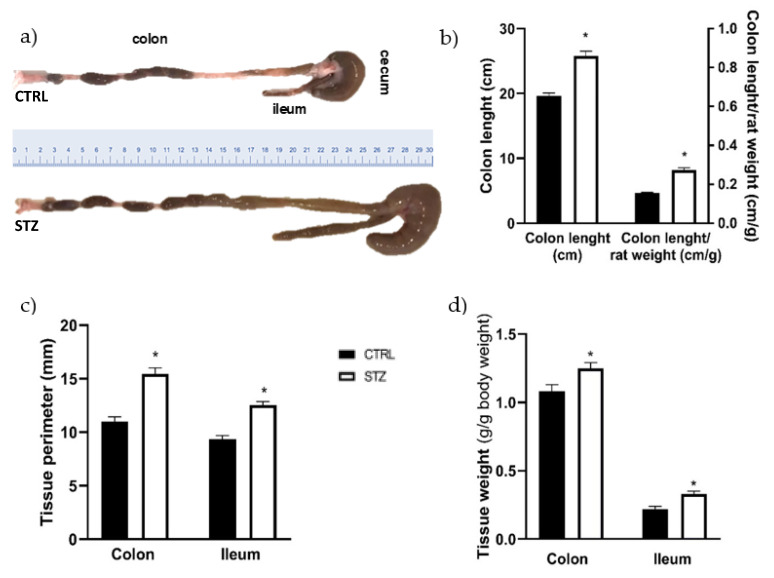
Macroscopic evaluation of the ileum and colon of control (CTRL, black bars, *n* = 8–12) and streptozotocin-induced diabetic rats (STZ, white bars, *n* = 11–14): (**a**) representative images of the colon length; (**b**) quantitative analysis of colon length (left *y* axis) and colon length *per* rat weight (right *y* axis); (**c**) tissue circumferential perimeter of the colon and ileum and (**d**) relative weight of intestinal segments (without fecal content) expressed as g of colon or ileum/g of body weight. Values are mean ± SEM and unpaired student’s *t* test was used to compare the two experimental groups (CTRL and STZ). * Statistical difference, *p* < 0.05.

Comparing to control animals, all segments of the intestines of STZ rats seemed enlarged. In addition, upon the opening of the abdomen of STZ-induced rats it was easy to perceive an extremely dilated cecum that produced a “mass effect”, pushing the intestine to the side. The colon length was significantly higher in diabetic animals compared to the control group (Figure 2a,b: 25.75 ± 0.77 cm, *n* = 14 vs. 19.63 ± 0.47 cm, *n* = 12, *p* < 0.05). Since some animals were heavier than others, colon length *per* body weight was measured and the difference between the two groups was maintained (Figure 2b). The circumferential perimeter of the intestinal portions was also measured, being significantly higher in the STZ-induced rats (*n* = 11) compared to non-diabetic rats (*n* = 8) both in the colon (15.45 ± 0.58 mm vs. 11 ± 0.46 mm, *p* < 0.0001, respectively) and ileum (12.55 ± 0.31 mm vs. 9.38 ± 0.32 mm, *p* < 0.0001, respectively) (Figure 2c). The relative weight of the whole intestine segment studied (with fecal content) was higher in STZ-induced animals than in controls (2.69 ± 0.10 g/g of body weight, *n* = 21 vs. 1.80 ± 0.05 g/g of body weight, *n* = 12; *p* < 0.0001, respectively). This increase was also observed at the individual intestinal segments free of fecal content (Figure 2d). Furthermore, no differences were found between STZ-induced animals and controls in the wet-to-dry ratio of all the segments studied (ileum: 5.23 ± 0.37 vs. 5.61 ± 0.33; PC: 5.17 ± 0.24 vs. 4.52 ± 0.20; MC: 4.84 ± 0.30 vs. 5.16 ± 0.21; DC: 5.07 ± 0.20 vs. 4.86 ± 0.28, respectively, *p* > 0.05 for all). The 2-way ANOVA results showed an interaction between the experimental group (control or STZ) and the intestinal segments (*p* < 0.0001), in accordance with our visual observation of the marked dilatation of the intestine in STZ-induced animals. The relative fecal content weight was also higher in STZ-induced animals than in controls (7.10 ± 0.15 g/g of body weight, *n* = 21 vs. 2.66 ± 0.11 g/g of body weight, *n* = 12; *p* < 0.0001). To our knowledge, this is the first time that the weight of intestinal content is reported in STZ rats. 

### 2.3. Ileum and Colon Microscopic Evaluation

**Figure 3:**
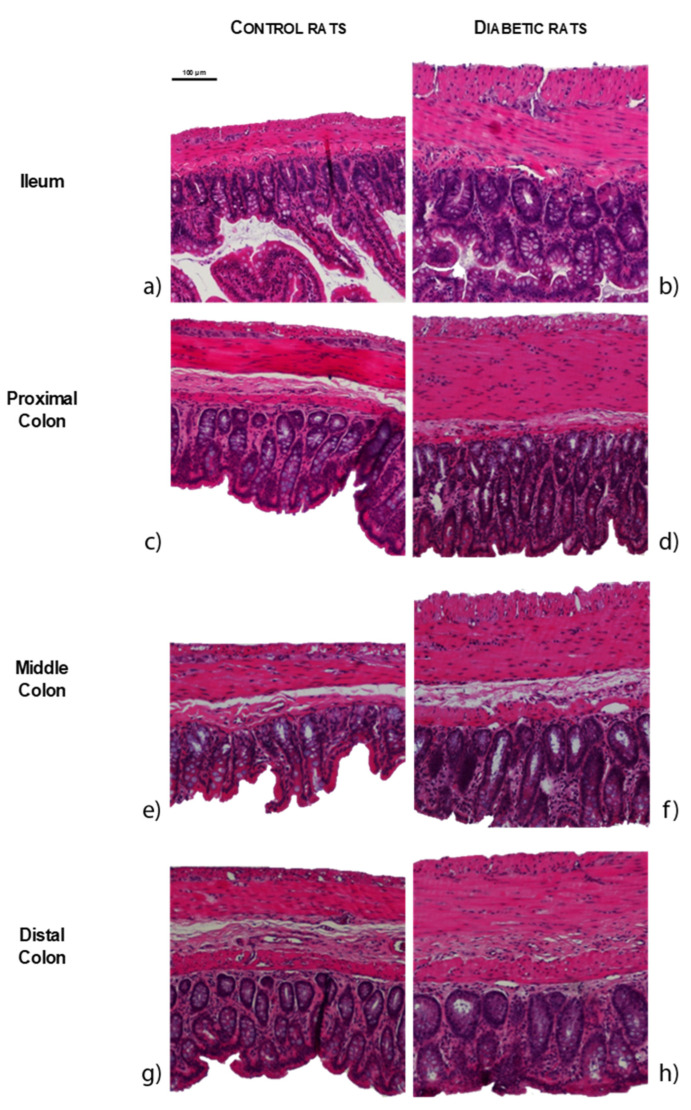
Representative microscopic photographs of intestinal segments of control (CTRL, **a**,**c**,**e**,**g**) and streptozotocin-induced diabetic rats (STZ, **b**,**d**,**f**,**h**), stained with hematoxylin and eosin: ileum (**a**,**b**); proximal colon (**c**,**d**); middle colon (**e**,**f**) and distal colon (**g**,**h**). The scale bar (100 µm) is valid for all images.

**Figure 4:**
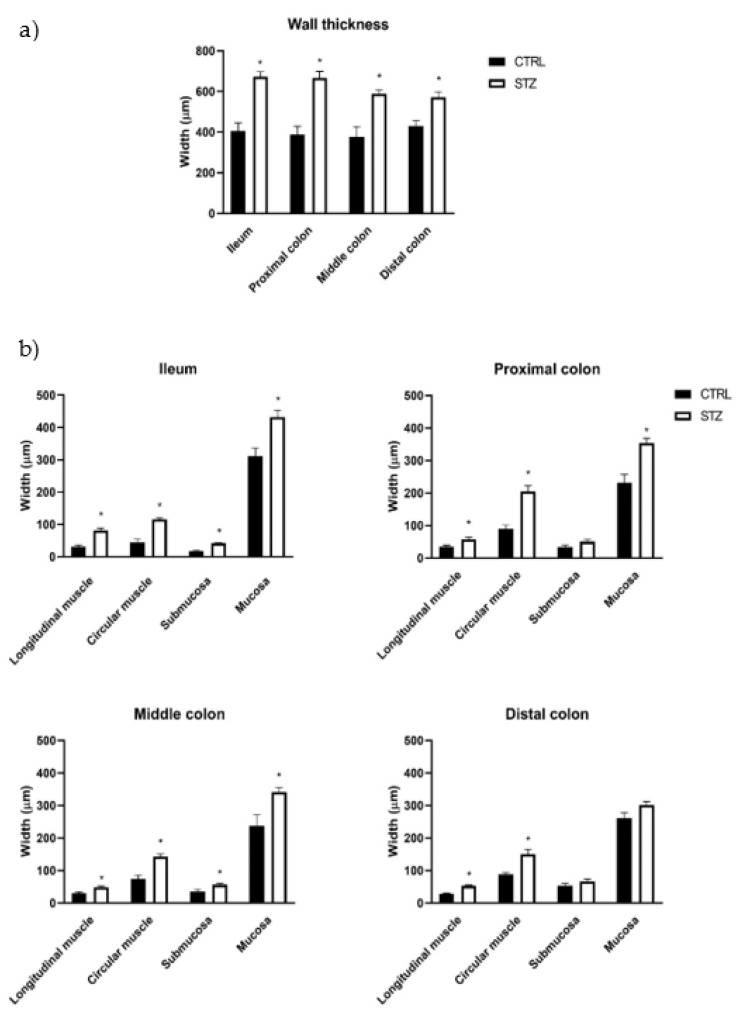
Morphometric evaluation of intestinal segments (ileum, proximal colon, middle colon and distal colon) of control (CTRL, *n* = 4) and streptozotocin-induced diabetic (STZ, *n* = 8) rats: (**a**) total wall thickness (μm) of each intestinal segment; (**b**) thickness (μm) of the intestinal layers (longitudinal muscle, circular muscle, submucosa and mucosa) of each intestinal segment. Values are mean ± SEM and a 2-way ANOVA followed by an unpaired t test with Welch’s correction was used to compare the two experimental groups (CTRL and STZ). * Statistical difference *p* < 0.05 vs. correspondent control.

The results of the histomorphometric evaluation of the intestines of STZ-induced animals (*n* = 8) were concordant with the macroscopic data, showing an increase in the thickness of the intestinal wall of the ileum, proximal colon (PC), middle colon (MC) and distal colon (DC) compared to controls (*n* = 4), as can be observed in Figure 3 and Figure 4a (ileum: 671.64 ± 74.34 µm vs. 404.97 ± 82.04 µm; PC: 666.66 ± 32.340 µm vs. 389.24 ± 39.03 µm; MC: 589.03 ± 17.88 µm vs. 376.06 ± 50.62 µm; DC: 570.93 ± 27.16µm vs. 430.42 ± 26.26µm, respectively, *p* < 0.01 for all). The intestinal wall thickness increase was similar for all the intestinal segments, as 2-way ANOVA showed a non-significant association (*p* = 0.1681) between experimental group and intestinal segment. Both ileum (longitudinal muscle: 81.02 ± 7.66 µm vs. 31.18 ± 5.44 µm, circular muscle: 116.12 ± 4.59 µm vs. 44.47 ± 10.40 µm, submucosa: 41.68 ± 1.68 µm vs. 17.47 ± 2.13 µm, mucosa: 432.82 ± 20.59 µm vs. 311.85 ± 24.51 µm, respectively, *p* < 0.01 for all) and middle colon (longitudinal muscle: 48.93 ± 2.93 µm vs. 29.66 ± 4.25 µm, circular muscle: 142.55 ± 8.37 µm vs. 74.31 ± 10.9 µm, submucosa: 56.39 ± 4.09 µm vs. 35.63 ± 6.47 µm, mucosa: 341.17 ± 13.79 µm vs. 236.46 ± 34.58 µm, respectively, *p* < 0.05 for all) presented increased thickness of all the intestinal layers assessed in STZ-induced rats compared to controls (Figure 4b). In the PC, the submucosa was the only layer that presented a similar thickness between STZ-induced animals and controls (50.47 ± 7.33 µm vs. 33.81 ± 6.00 µm, respectively, *p* = 0.1104), while all the other segments were thicker in diabetic animals compared to controls (longitudinal muscle: 57.02 ± 6.90 µm vs. 34.64 ± 4.29 µm, circular muscle: 205.2 ± 17.00 µm vs. 90.14 ± 11.33 µm, mucosa: 353.97 ± 14.27 µm vs. 230.64 ± 26.18 µm, respectively, *p* < 0.05 for all). DC only showed an increase in the muscle thickness (longitudinal muscle: 52.51 ± 2.72 µm vs. 28.51 ± 1.67 µm, circular muscle: 150.54 ± 14.58 µm vs. 87.21 ± 7.06 µm, *p* < 0.01 for both; submucosa: 66.11 ± 7.70 µm vs. 53.27 ± 7.54 µm and mucosa: 301.77 ± 10.00 µm vs. 261.42 ± 16.49 µm, *p* > 0.05 for both) (Figure 4b). The 2-way ANOVA showed an association between the experimental group (control vs. STZ) and the intestinal layers thickness (longitudinal muscle, circular muscle, submucosa and mucosa) for the ileum (*p* = 0.0058), PC (*p* = 0.0002), MC (*p* = 0.0027) but not for the DC (*p* = 0.1109).

### 2.4. Ileum and Colon Functional Evaluation

**Figure 5:**
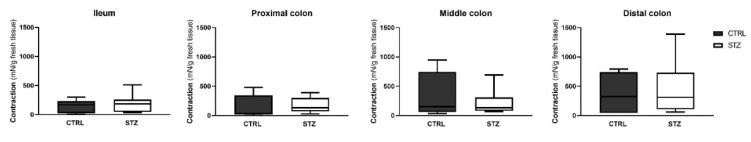
Contractile response to KCl (125 mM) in the ileum, proximal colon, middle colon and distal colon of control (CTRL, *n* = 6) and streptozotocin-induced diabetic rats (STZ, *n* = 10). Data are expressed as mN of force *per* g of fresh tissue (mN/g). Values represent the median (95% confidence limits) and a Mann–Whitney test was used to compare the two experimental groups (CTRL and STZ).

**Figure 6:**
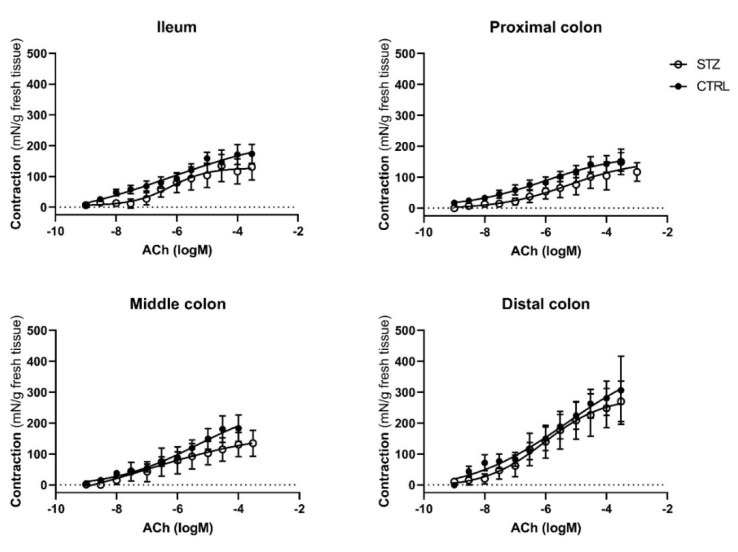
Concentration-response curves to ACh in the ileum, proximal colon, middle colon and distal colon of control (CTRL, *n* = 6–7) and streptozotocin-induced diabetic rats (STZ, *n* = 10). Data are expressed as mN of force per g of fresh tissue (mN/g). Values are mean ± SEM.

**Figure 7:**
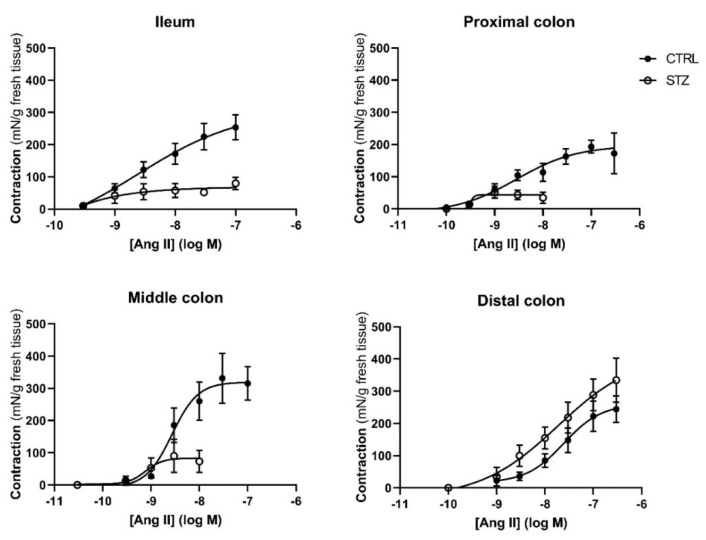
Concentration-response curves to Angiotensin II in the ileum, proximal colon, middle colon and distal colon of control (CTRL, *n* = 5–8) and streptozotocin-induced diabetic rats (STZ, *n* = 5). Data are expressed as mN of force *per* g of fresh tissue (mN/g). Values are mean ± SEM.

To assess whether intestinal muscle contraction is altered in diabetic animals, ileum and colon reactivity to exogenously applied KCl (Figure 5), ACh (Figure 6) and Ang II (Figure 7) was evaluated. For the concentration–response curves to ACh and Ang II the results were expressed using two recognized pharmacological concepts: the maximum contractile effect (E_max_, expressed in mN/g) and the concentration of agonist capable of causing 50% of the maximal contraction (EC_50_, expressed in uM). In all intestinal segments (ileum, PC, MC and DC) the contractile response to 125 mM KCl (and the ACh concentration-dependent contraction were similar in both control and STZ-induced animals (Figure 6), with comparable E_max_ and EC_50_ values, presented in Table 1.

Regarding reactivity to Ang II, this RAS effector peptide caused a concentration-dependent contraction in control and diabetic animals (Figure 7). The contractile response to Ang II normalized to the tissue weight was lower (but with the same EC_50_) in the ileum, PC and MC of STZ-induced animals. Interestingly, the maximum response in the DC was similar between control and STZ-induced animals, but the EC_50_ of that portion of diabetic colon was significantly lower than that of controls (Table 2). 

**Figure 8:**
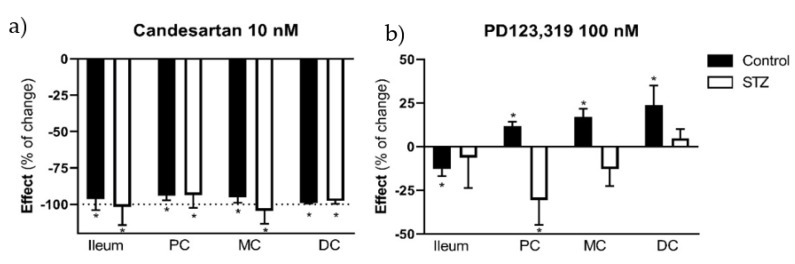
Angiotensin II contractile effect (expressed as percentage of change) in the ileum, proximal colon (PC), middle colon (MC) and distal colon (DC) of control (CTRL, *n* = 5–8) and streptozotocin-induced diabetic rats (STZ, *n* = 4–6) in the presence of the following antagonists: (**a**) candesartan (AT_1_R antagonist, 10 nM) and (**b**) PD123,319 (AT_2_R antagonist, 100 nM). Values are mean ± SEM. For statistical analysis we used a paired t test between the effect in the absence and presence of the antagonist.* *p* < 0.05 vs. the correspondent response to Angiotensin II in the absence of the antagonist.

Knowing that the differences observed in the contractile response to Ang II could result from an imbalance between AT_1_R and AT_2_R mediated effect, we decided to further characterize the response to Ang II. The contractile response to Ang II was antagonized by candesartan (10 nM), an AT_1_R antagonist, in all four intestinal segments of both control (in mN/g for all, ileum: 54.20 ± 4.50 vs. 2.35 ± 1,60; PC: 17.37 ± 3.14 vs. 1.07 ± 0.49; MC: 12.42 ± 2.23 vs. 0.28 ± 0.15; DC:15.85 ± 1.32 vs. 0.1 6± 0.08; *p* < 0.05 for all) and STZ-induced rats (ileum: 35,75 ± 11,06 vs. −0,87 ± 2,78; PC: 24.80 ± 9.45 vs. 0.7 8± 1.19; MC :95.86 ± 29.03 vs. 5.20 ± 6.39; DC: 288.48 ± 49.08 vs. 5.57 ± 5.54; *p* < 0.05 for all) (Figure 8a). Differently, PD123319 (AT_2_R antagonist, 100 nM) decreased the response to Ang II in the ileum (12.43 ± 1.03 mN/g vs. 11.02 ± 1.21 mN/g, *p* < 0.05) and increased the response in all colonic segments of control animals (in mN/g for all, PC: 19.95 ± 3.34 vs. 22.02 ± 3.45; MC: 14.99 ± 1.97 vs. 17.48 ± 2.44; DC: 19.88 ± 2.82 vs. 23.50 mN/g ± 2.64; *p* < 0.05 for all), but was unable to modify Ang II-induced contraction in the ileum (92.58 ± 21.23 mN/g vs. 104.24 ± 23.50 mN/g), MC (146.13 ± 18.53 mN/g vs. 127.88 ± 21.89 mN/g) and DC of diabetic rats (236.37 ± 19.03 mN/g vs. 248.38 ± 25.64 mN/g; *p* > 0.05 for all), decreasing it in the PC (166.14 ± 20.49 vs. 108.45 ± 19.00; *p* < 0.05) (Figure 8b).

## 3. Discussion

Our data show that the diabetic rat model chosen (DM chemically induced by an IP STZ injection, maintained for 14 days) presented all the typical signs of T1DM: body weight loss, polyphagia, polyuria and polydipsia [33,34,35,36,37]. In addition, diabetic rats gradually increased their fecal excretion whereas non-diabetic animals maintained a relatively stable fecal excretion during the entire experimental period. As pointed out before, this is the first study to quantify fecal excretion in STZ-induced diabetic animals. Besides the increase in mass, the fecal pellets from the diabetic group were well formed but were larger, wider and darker than those from the control group. These findings could eventually be attributed to polyphagia and intestinal distension, differing from Cuervas–Mon and collaborators’ data, who described STZ-induced diabetic rats’ feces as thick and amorphous, compared to those of control animals [38].

To our knowledge, this study is the first to show that the colon length and the perimeter of the ileum and colon are increased in this early DM model, and that the differences between control and STZ correlate to the different portions studied, in accordance with our visual observation of the marked dilatation of the intestine in STZ-induced animals.

Indeed, enlargement and increased length of the intestine and colon of STZ rats were already described by others, 10 and 8 weeks after DM induction, respectively [39,40]. A possible explanation for the increase in colon and intestine length described in these studies is the remodeling of the extracellular matrix (due to increased production of collagen type 1) and AGE accumulation [40]. In this study we decided to measure only the length of the colon, as it is macroscopically difficult to distinguish ileum boundaries. Our data also show that just 2 weeks after induction, STZ-induced rats present an increase in ileum and colon weight. Forrest and colleagues found that dry colon weight increased significantly in diabetic animals (8 weeks after induction) compared to controls and suggested that this could be related to increased colon length, since weight *per* length did not differ between the two experimental groups [35]. Others observed that weight, but not length, of insulin-treated diabetic rats was significantly higher compared to controls, thus contradicting Forrest and collaborators [41,42]. A possible explanation for the intestinal wall weight increase may be related to the tissue water content, which has been reported to be higher in diabetic animals [39]. However, we did not observe any difference between control and STZ-induced animals in the wet-to-dry ratio of the intestinal segments studied, results that are corroborated by other researchers [35]. For the time being, there is no clear answer as to which mechanisms are triggering the intestinal mass increase in diabetic animals, but Jervis and colleagues suggested that this enlargement could be an adaptation to polyphagia, a characteristic sign of the disease, since intestinal smooth muscle cells are plastic and adapt to functional demand, by remodeling [43]. Curiously, other causes of polyphagia such as lactation or hypothalamic lesions seem to induce similar intestinal consequences [44,45,46]. On the other hand, another study revealed that even when the food intake of diabetic rats was matched to that of controls, the intestinal weight of diabetic animals remained higher [9]. 

Our study innovatively uncovers several early histomorphometric alterations in the ileum and colon of T1DM rats and these alterations did not differ according to the different portions studied. Indeed, there are no previous histopathological data on the colon of STZ-induced rats just 2 weeks after induction, although a previous study showed similar results in the ileum 7 and 14 days after induction [9]. The same authors also studied histological characteristics of the middle colon, reporting increased intestinal wall thickness in longer STZ-induced models (4 and 8 weeks after induction) compared to controls [10].

Contrary to what happens when we look at the intestinal wall as a whole, the differences seen by layers are determined by the portion studied. This occurs since in the distal colon only the muscle layers are affected. So, the variation in thickness of the layers of the intestinal wall between diabetic and control animals becomes progressively less evident in the proximal–distal direction (from ileum to distal colon), in agreement with what was previously described by Fregonesi and collaborators [18]. This is a curious finding that reinforces the relevance of studying several intestinal segments to avoid generalizing phenomena that may occur in specific regions. Several studies indicate that increased intestinal thickness in diabetic animals may be due to: a) increased mucosa proliferation (due to higher food intake, increased expression of glucagon-like peptide 2, accumulation of AGE and/or suppression of apoptosis) and b) increased muscle layers (due to AGE mediated effects, collagen type I accumulation and/or smooth muscle cells hypertrophy) [33,40,47,48,49,50,51]. However, further studies are needed to understand if any of the possibilities mentioned above explain the histomorphometric alterations observed, or if there are other mechanisms involved.

The studies conducted on ileum and colon reactivity suggest that there are no changes in the intestinal function of STZ-induced rats just two weeks after induction, since the contractile response to KCl and ACh remained unchanged in all segments studied. Previous studies using rat ileum showed a decrease in the contractile response to ACh 30 days and 6 months after STZ-induction, but this change does not seem to be related to cholinergic innervation damage or acetylcholinesterase activity modification [21,38,52]. Concerning the colon, it was not possible to find differences between the contractile response to ACh in control and STZ-induced rats, injected 30 days previously [52]. However, in a genetic model of T2DM, after a long period of disease (60 weeks) the contractile response to carbachol (an ACh mimetic) in the PC was lower than that of controls, while the response in the DC appeared to be unaffected [22]. Thus, it seems that cholinergic activity in the colon and ileum of diabetic animals may depend on several factors, such as type of diabetes, intestinal segment affected and diabetes evolution time, suggesting that main alterations in diabetic intestinal motility are probably related to changes in smooth muscle layers and non-cholinergic innervation [21,22,38]. 

We therefore decided, in an innovative way, to evaluate the reactivity of the ileum and colon of diabetic animals to Ang II. The results presented in the functional studies suggest a loss of contractile force in response to Ang II in the ileum, PC and MC but not in the DC of STZ-induced rats, compared to controls, probably due to the fact that the distal segments of the GI tract are the last ones to be affected by diabetic complications [18]. To our knowledge this is the first time that an altered Ang II response is reported in diabetic animals, an effect that could be associated with the structural alterations observed, loss of specific neurons (mostly in the myenteric plexus) and changes in the local tissue levels of Ang II [17,27,53]. Ang II activates both receptors in the smooth muscle cells but also presynaptic receptors in other cells crucial for colonic function, an intricate network that has been reported to be altered in the diseased colon [27,29,54]. Regarding Ang II-mediated effects, it is known that contractile responses in intestinal smooth muscle occur mainly through the activation of AT_1_R, while AT_2_R’s role according to our group and others, seems to be more important under pathological conditions [27,55,56]. Not surprisingly, we observed that the AT_1_R antagonist (candesartan, 10 nM) completely abolished AngII-mediated contractile response in the ileum and all colon segments of both control and diabetic animals. However, the blockade of AT_2_R with PD123319 (100 nM) was more intriguing. In the colon of control rats, we observed that the AT_2_R-associated counterbalance of Ang II AT_1_R-mediated contractile effects was no longer present in the DC and MC of diabetic animals, and was even reversed in the PC, as we have reviewed previously [53]. Interestingly, the contractile effect of Ang II in the ileum of control rats was decreased in the presence of PD123319. This points to a putative contractile effect mediated by the AT_2_R, which although uncommon was previously described in other studies [57,58]. Even so, in the ileum this is not observed, reinforcing the idea that under pathological conditions the effect mediated by the AT_2_R in the ileum and throughout the colon is loss/altered, as previously described by our group in an experimental model of colitis in rats [27,53,59].

## 4. Materials and Methods

### 4.1. Animals and Housing

Since female rats seem to be less sensitive to STZ [7], forty-seven male Wistar rats, 10 to 14 weeks in age (weighing 300–400 g), were used in this study, including control (*n* = 24) and diabetic (*n* = 23) animals, that were distributed between the different experimental protocols. All control animals were used in the experimental procedures (since we used the same intestinal portions in different functional studies), but only eight of these rats were daily monitored in the animal house facility. Control animals were used in collaboration with other groups that collected organs such as heart, muscle and brain, in a perspective of reducing animals used in experimental research. Sample size was decided using the free software Sample Size Calculator (^©^2022—ClinCalc LLC, https://clincalc.com/stats/samplesize.aspx). Animals were maintained at the ICBAS-UP rodent animal house facility and the project was approved by the animal welfare body (P311/2019). This work followed the ARRIVE guidelines for reporting experiments with animals [60] (see supplementary material). Animals were maintained in a 12 hours’ light/dark cycle, with controlled ventilation, temperature (20–24 °C) and relative humidity (40–60%). All animals were housed in groups of two in Sealsafe Plus GR900 Tecniplast^®^ cages with proper bedding (Corncob ultra 12, Ultragene), with free access to autoclaved tap water (two bottles per cage) and laboratory rodent food (4 RF21, Mucedola S.r.l., Italy). Environmental enrichment such as paper tunnels and nesting material was provided in all animal cages.

### 4.2. Diabetes Induction

On the day of DM induction (d0) animals were fasted for 4 h (food taken from the box where the animals were housed) with free access to water. The STZ solution (S0130, Sigma-Aldrich, St. Louis, MO, USA; 55 mg/mL in citrate buffer, pH 4.5) was prepared just prior to the injection, since a freshly prepared solution is considered to be more effective [4]. Diabetes was randomly induced by a single intraperitoneal injection of 55 mg/kg of STZ (a concentration that has proven successful in our group (data not published) and also by other authors [61]), under the analgesic effect of tramadol (Tramal^®^ oral suspension, 100 mg tramadol/mL, Grünenthal, Portugal) (20 mg/kg, PO), administered moments before [4]. The total volume of STZ solution (55 mg/kg) administered to each animal depended on its weight on the day of induction, ranging from 0.3 to 0.4 mL. Rats had *ad libitum* access to water and food until the end of the protocol (day 14). Animals were considered diabetic if 48 h after STZ injection their blood glucose was ≥250 mg/dL, a situation that occurred in 23 of the 32 animal that were induced (diabetes induction success of 72%). These 23 hyperglycemic rats were included in the STZ group and used in the respective experimental protocols. Glycemia was evaluated using a FreeStyle Precision Neo (Abbott, Canada) glucometer. The blood glucose level of diabetic rats was measured by puncturing one of the tail veins at d0 (control value), d2 (to confirm or discard DM) and d7. On d14, animals were sacrificed by decapitation, using a guillotine suitable for rats (Small Guillotine, Harvard Apparatus) and blood glucose levels were obtained from blood samples collected from the abdominal aorta. 

### 4.3. Animal Monitorization and Welfare Evaluation 

The animals included in this project were daily monitored (11:00 h to 13:00 h) throughout the entire protocol (d0–d14), and all information was registered in an individual evaluation table (confounders were not controlled). The evaluation started in the maintenance room, assessing the coat’s appearance, piloerection, animal’s posture, abdominal discomfort and changes in the breathing pattern (welfare evaluation). Then, in the observation room and with the cage open, the same parameters were observed, and the animals’ hydration status was evaluated. Monitoring proceeded by weighing the animal and water/food in order to calculate daily intake. The appearance of the feces was also evaluated, and fecal pellets were weighed 48 h after collection to assure uniform drying of all collected samples. The cages were changed every 2 days or whenever they became excessively wet due to diabetes-associated polyuria.

### 4.4. Intestinal Macroscopic Evaluation

On protocol d14 control and STZ-induced rats were euthanized. The abdomen was opened, and the overall appearance of the viscera was evaluated. The abdominal aorta was identified and punctured to collect blood to measure glycemia. The ileum and colon were collected and weighed intact and after cleaning gently their content using Krebs-Henseleit solution (in mM: 118 NaCl; 4.8 KCl; 2.5 CaCl_2_·2H_2_O; 1.2 NaH_2_PO_4_·H_2_O; 1.2 MgSO_4_·7H_2_O; 25 NaHCO_3_; 0.02 Na_2_EDTA; 0.3 Ascorbic acid; 11 monohydrated glucose). The longitudinal length of the colon was measured and a 1 cm portion of the ileum and middle colon was opened through the non-mesenteric border and laid flat to measure the circumferential perimeter (mm).

### 4.5. Intestinal Microscopic Evaluation

Samples (0.5 cm long) of the ileum and colon of diabetic and control animals were collected for histological examination. More precisely, the portion of the ileum was collected 3 cm proximal to the ileocecal junction; the proximal colon (PC) was collected 3 cm distal from the cecum; the distal colon (DC) 3 cm proximal to the anus and the middle colon (MC) 3 cm proximal to where the DC was collected. Each sample was opened through the anti-mesenteric border and fixed in 4% formalin. Samples were routinely processed and paraffin-embedded, cut in 3 µm-thick sections and stained with hematoxylin-eosin (HE) for histological evaluation [10]. Each section was evaluated under an optical microscope (Nikon, model Eclipse E600, Nikon Instruments, Miami, FL, USA) and photographed in two or three different representative regions with objective lens of 4×, 10× and 20× (magnification of 40×, 100× and 200×). The images were used to measure the thickness of the mucosa, submucosa, circular muscle and longitudinal muscle, always by the same person, using the free ImageJ^®^ software 1.53t. For each sample the layer thickness was measured in nine different locations and averaged. The measurements were only carried out in images where all the intestinal wall could be observed. 

### 4.6. Intestinal Functional Evaluation 

Four 1 cm long portions were collected from the ileum and colon of diabetic and control animals to evaluate smooth muscle contraction. The ileum was taken 2 cm proximal to the ileocecal junction; the PC 2 cm distal from cecum; DC 2 cm from anus and MC 2 cm proximal to the DC. Each sample was mounted in a vertical organ bath along its longitudinal axis, fixed to the bottom of the bath and to an isometric transducer (UGO BASILE S.R.I., Italy, Model 7004) using sewing threads. The bath was continuously aerated with carbogen (95% O_2_ and 5% CO_2_) and maintained at 37 ± 1 °C. Tissues were stretched to an initial resting tension of 1 g and mechanical responses were recorded using a PowerLab system (ADInstruments, Oxford, UK). All tissues were washed twice, every 15 min, and triggered with 10 µM of ACh. They were then washed and allowed to stabilize for 15 min more before starting one of the following protocols:

- a cumulative concentration–response curve to ACh (Sigma-Aldrich, USA; 1 nM to 10 mM)
- a non-cumulative concentration–response curve to Ang II (Sigma-Aldrich, USA), according to the range of concentrations that was previously determined in other studies of this research group: ileum, PC and MC: 300 pM to 100 nM; DC: 1 nM to 300 nM [27]. Between each Ang II concentration tissues were washed for 1 h (every 15 min), to avoid receptor desensitization.
- the response to a single concentration of Ang II (Ileum, PC and MC: 30 nM, DC: 100 nM) in the absence and presence of candesartan (a kind gift from Dr. Fredrik Palm, Uppsala University, Sweden; 10 nM, AT_1_R antagonist) or PD123319 (Sigma-Aldrich, USA; 100 nM, AT_2_R antagonist). Tissues were incubated for 20 min with the antagonists before the second stimulation with Ang II.

At the end of every protocol, the contractile response to potassium chloride (KCl, 125 mM) was recorded. 

Finally, each portion used in the functional study was weighed immediately after the protocol (fresh weight) and after drying for 48 h, at room temperature (dry weight). The fresh weight was used to normalize the contractile response. Fresh and dry weight were used to calculate the wet-to-dry ratio, as an index of edema, according to the following equation: WtDr = (WetWeight − DryWeight)/DryWeight.

### 4.7. Statistical Analysis 

The GraphPad Prism^©^8.1.2 software was used for statistical analysis of data. The unpaired Student’s t-test was used to analyze animal monitorization and macroscopic evaluation. For comparison between two experimental groups (CTRL and STZ) the Student’s t test was used for variables with a Gaussian distribution and the Mann–Whitney test for those with a non-Gaussian distribution. The two-way ANOVA was used to look for interaction in the data from histological evaluation and functional data. Accordingly, data were expressed as mean ± SEM for the Student’s *t*-test and median [95% CI] for the Mann–Whitney test where “*n*” indicates the number of animals per group. In all cases, a *p* value of less than 0.05 was considered to denote a statistically significant difference. 

## 5. Conclusions

**Figure 9:**
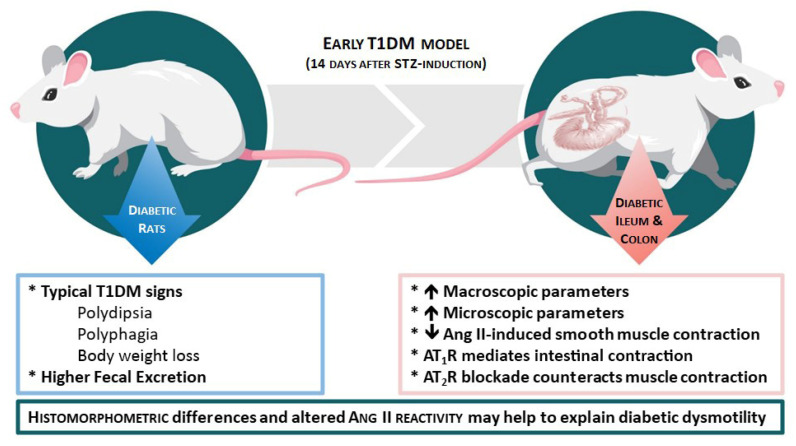
Schematic representation of the major findings observed in this early T1DM model. Diabetic rats showed typical DM signs (**left** part of the scheme). The ileum and colon revealed an increase in both macro/microscopic parameters and a decrease in Ang II-induced smooth muscles contraction, mediated by both AT_1_R and AT_2_R activation (**right** part of the scheme).

The results presented in this study demonstrate that it is possible to refine a classic animal model of T1DM, improving animal welfare. In this early (two-week evolution) STZ-induced T1DM model we observed (Figure 9): (1) all the characteristic signs of T1DM (polydipsia, polyuria, polyphagia and body weight loss) and increased fecal excretion; (2) increased length, perimeter and weight in the ileum and colon; (3) increased thickness of several histological intestinal layers (less evident in CD) of the ileum and colon, and (4) decreased Ang II-induced smooth muscle contraction (less evident in the DC) associated with altered balance between the function of Ang II receptors. These reported histomorphometric differences and altered reactivity may help to explain diabetic enteric dysmotility and will be deepened in future studies. 

## Figures and Tables

**Table 1:** E_max_ (mN/g) and EC_50_ (μM) values of smooth muscle contraction induced by ACh application in the ileum, proximal colon, middle colon and distal colon of control (CTRL, *n* = 6–7) and streptozotocin-induced diabetic rats (STZ, *n* = 10).

	Ileum	Proximal Colon	Middle Colon	Distal Colon
Control				
E_max_ (mN/g)	165.9 [116.4–216.0]	141.0 [116.7–278.5]	184.7 [68.95–378.8]	313.4 [176.2–823.1]
EC_50_ (μM)	0.85 [0.32–3.53]	1.15 [0.22–14.70]	3.41 [1.1–4.8]	2.74 [0.94–7.47]
STZ				
E_max_ (mN/g)	79.06 [34.65–338.9]	158.0 [75.0–569.5]	143.6 [86.56–411.3]	271.7 [163.6–370.9]
EC_50_ (μM)	0.82 [0.27–1.87]	114.0 [8.31–3408]	18.96 [0.87–75.7]	2.94 [0.28–142.0]

For comparison between the two experimental groups (CTRL and STZ) we used a Mann–Whitney test. Values are median (95% confidence limits).

**Table 2:** E_max_ (mN/g) and EC_50_ (μM) values of smooth muscle contraction induced by Angiotensin II application in the ileum, proximal colon, middle colon, and distal colon of control (CTRL, *n* = 5–8) and streptozotocin-induced diabetic rats (STZ, *n* = 5).

	Ileum	Proximal Colon	Middle Colon	Distal Colon
Control				
E_max_ (mN/g)	305.3 [138.6–620.5]	181.5 [136.0–297.0]	276.6 [246.4–451.1]	344.4 [222.4–433.5]
EC_50_ (μM)	8.29 [1.24–24.68]	1.10 [0.36–2.12]	3.80 [1.95–4.76]	40.50 [17.08–309.3]
STZ				
E_max_ (mN/g)	71.20 [12.3–100.6] *	50.46 [15.32–78.15] *	100.6 [22.86–163.5] *	263.5 [165.0–415.9]
EC_50_ (μM)	7.985 [0.31–8.89]	0.59 [0.35–14.93]	2.60 [0.89–7.81]	4.17 [0.84–8.38] *

For comparison between the two experimental groups (CTRL and STZ) we used a Mann–Whitney test. Values are median (95% confidence limits). * *p* < 0.05 vs. correspondent control.

## Data Availability

Data is available upon request.

## Supplementary Materials

The supporting information can be downloaded at: https://www.mdpi.com/article/10.3390/ijms232113233/s1
